# Involvement of Specialist Palliative Care in a Stroke Unit in Austria—Challenges for Families and Stroke Teams

**DOI:** 10.3389/fneur.2021.683624

**Published:** 2021-09-22

**Authors:** Renate Riesinger, Klaus Altmann, Stefan Lorenzl

**Affiliations:** ^1^Department of Palliative Care, Hospital Barmherzige Schwestern, Ried im Innkreis, Austria; ^2^Department of Neurology, Hospital Barmherzige Schwestern, Ried im Innkreis, Austria; ^3^Institute of Nursing Science and Practice, Paracelsus Medical University, Salzburg, Austria; ^4^Ludwig-Maximilians-University, Munich, Germany; ^5^Department of Neurology, Klinikum Agatharied, Hausham, Germany

**Keywords:** early integration, patient care planning, family satisfaction, FS-ICU 24, decision making, ethics, severe stroke, specialist palliative care

## Abstract

**Purpose:** Severe stroke poses vast challenges. Appropriate goals of care according to individual preferences and values have to be developed under time restrictions—often impeded by limited ability to communicate and the need for decisions by surrogates. The aim of our study was to explore the decision-making process and the involvement of specialist palliative care in the acute phase of severe stroke.

**Methods:** Twenty patients suffering from severe ischemic stroke treated in an Austrian acute inpatient stroke unit were included in a prospective study. Their families were interviewed with a questionnaire (FS-ICU 24), which covered satisfaction with care and decision-making. With a second questionnaire, decision-making processes within the stroke team were investigated.

**Results:** A palliative approach and early integration of specialist palliative care in severe ischemic stroke results in individualized therapeutic goals, including withholding therapeutic or life-sustaining measures, especially in patients with pre-existing illness.

**Conclusions:** Family members benefit from understandable and consistent information, emotional support, and a professional team identifying their needs. Stroke unit professionals need skills as well as knowledge and strategies in order to make decisions and provide treatment at the end-of-life, when there may be ethical or legal issues. Close cooperation with specialist palliative care services supports both treatment teams and families with communication and decision-making for patients with severe ischemic stroke.

## Introduction

Stroke is a leading cause of death and disability worldwide. Despite a decline in incidence and mortality in recent years, the prevalence of stroke increases due to a growing and aging population. Therefore, stroke will remain a major concern globally (1).

American and Canadian professional societies recommend palliative care as an integrated part of stroke care (2, 3). When stroke affects activities and quality of life and reduces life expectancy, patients and their families should have comprehensive access to palliative care from the moment of diagnosis and throughout the entire course of the illness, particularly in the presence of progressive chronic comorbidities or preexisting palliative care goals (3).

In severe stroke, families are confronted with an acute onset and the victim's devastating decline in function and cognition—often accompanied by loss of verbal communication skills. Prognosis on the course and outcome is often unclear. Anxiety and depression among stroke patient's family members are common (4) and the related emotional burden is also measurable 1 year after the incident (5).

In this situation, preference-sensitive decisions need to be made. Advance directives are rare and patient's autonomy is often determined via proxies who base their opinion on previously expressed wishes of the patient or give advice in the best interest of the patient (6). Although decision-making can entail enormous emotional burden, caregivers want to be involved (7, 8). However, the surrogate's decisions can be influenced by culture and religion, cognitive biases (3), as well as by her/his own wishes and values (9).

Decision-making, alignment of treatment with the patient's goals, emotional support for families, and the basics of symptom management are core elements of palliative care and should be routine aspects of care for anyone caring for stroke patients and their families (3).

The aim of our study was to assess whether the approach of having early and close cooperation with specialist palliative care (SPC) services has an impact on stroke patient's families sense of satisfaction. Furthermore, we intended to evaluate decision-making in the context of limiting life-sustaining therapies (LST) in severe stroke.

## Methods

Patients with severe ischemic stroke admitted to the acute stroke unit of Barmherzige Schwestern in Ried im Innkreis, a secondary/tertiary care hospital in Austria, Europe, between June 2019 and February 2020 were studied.

Inclusion criteria were severe ischemic stroke (modified Rankin Scale ≥4 after acute therapy or no therapeutic options), age ≥18 years, contact to a palliative care team during hospital stay, and visit by family member(s) during stroke unit stay.

The palliative care team, including a palliative care physician and nurse, was involved following the decision of the treating physician, without the use of predefined triggers for referral.

Data about patients were collected prospectively during the study period and data on the role and involvement of the palliative care team retrospectively using patient's medical charts. Data analyses were descriptive in nature.

For the study of stroke patients' relatives, the questionnaire FS-ICU 24 was used. This questionnaire is available in German and assesses family satisfaction with care and decision-making in a critical care setting (10). The researcher contacted them in person or via telephone within 1–4 weeks after the patient's discharge from the stroke unit. As the study involved older participants, the survey was carried out on paper. Questionnaires were handed out, sent by mail or e-mail. If necessary, a reminder (e-mail or telephone call) was sent after 4 weeks. Questionnaires were handed in within 2 weeks to 2 months after the stroke unit stay.

The second part of the survey studied the stroke team's approach to limiting LST in all their patients, mostly stroke victims. In November and December 2019, team members (12 physicians and 20 nurses) were questioned via an electronic questionnaire based on a questionnaire previously used by Jox et al. in German intensive care units (ICU) (11). Participants were approached by e-mail. To maximize the response rate, an e-mail reminder was sent after 2 weeks.

The study was approved by the local ethics commission on June11, 2019.

## Results

### Patient Characteristics

A total of 427 patients with ischemic stroke were treated in this stroke unit in 2019. We identified 20 patients who received SPC during the 9-month-long study period, representing 5% of all patients admitted.

Patient characteristics can be found in Table 1.

**Table 1:** Patient characteristics.

	**Mean ± SD**	***%*, (*n*)**	
Age, years	83 ± 9		
65–74 75–84 ≥85		15 (3) 35 (7) 50 (10)	
Gender female		55 (11)	
Ethnicity		100 (20)	Caucasian
Stroke location		90 (18) 10 (2)	Anterior circulation Posterior circulation
Etiology		50 (10) 25 (5) 20 (1) 20 (1)	Cardioembolic Macroangiopatic Microangiopatic Unknown
mRS^a^ premorbid	3 ± 1		
mRS admission	5 ± 0		
NIHSS^b^	15 ± 6		
Comorbidities		80 (16) 55 (11) 45 (9) 40 (8) 20 (4) 10 (2) 5 (1) 5 (1) 5 (1)	Cardiac diseases Atrial fibrillation Dementia Heart failure Diabetes Heart attack in history Cancer Hemodialysis Smoking
PREMISE score (12)	8 ± 2		

a *mRS, modified Rankin Scale*.

b *NIHSS, National Institute of Health Stroke Scale*.

The mean PREMISE score, which predicts mortality within the first week after admission to a stroke unit (12) was 8 (±2) for all reviewed patients. This would imply a 19% mortality within the first week.

Therapies and complications can be seen in Table 2. It also shows that all patients had therapy limitations, introduced step by step during the stay. Due to the severity of stroke and concurrent reduced consciousness and/or comorbidities such as dementia, 17 patients (85%) were incapable of making decisions on their own. Two patients refused intensive care measures and cardiopulmonary resuscitation (CPR); one referred to her advance directive where she had refused CPR. Two patients (10%) had an advance directive.

**Table 2:** Therapies and course of illness.

		**%, (*n*)**	**Time (days) Mean ± SD**
**Therapies**			
	Intravenous thrombolysis	60 (12)	
	Thrombectomy	5 (1)	
	Craniectomy	0	
	Tracheotomy	0	
	PEG^a^ insertion	0	
**Complications**			
	Infection + antibiotics	35 (7)	
	Hemorrhage total Hemorrhage after iv thrombolysis	30 (6) 42 (5)	
**Decision making**			
	Capable of decision making	15 (3)	
	Advance Directive	10 (2)	
**Therapy limitations**			
Do Not Resuscitate (DNR)-*time till*		100 (20)	1 ± 2
Do Not Escalate (DNE)-*time till*		70 (14)	4 ± 7
Comfort Terminal Care (CTC)-*time till*		55 (11)	5 ± 8
**Stay**			
	Stroke unit		3 ± 3
	Hospital stay surviving		22 ± 11
	Hospital stay deceased		11 ± 11
**Referral from stroke unit**			
	Neurologic ward	45 (9)	
	Palliative care unit	45 (9)	
**Death**			
	Stroke unit	10 (2)	
	Overall deaths	60 (12)	
	Death after thrombolysis	58 (7)	

a *PEG percutaneous endoscopic gastrostomy*.

Most patients were transferred from the stroke unit. Overall, 60% died during the hospital stay.

SPC consultation took place 3 days (±3 days; mean ± SD) after stroke and, in 95% of cases, was conducted face-to-face. In one case, only telephone contact was made. On average, two contacts (±2; mean ± SD) occurred during the hospital stay. The palliative care physician was involved in all and the palliative care nurse in 35% of cases. The focus of palliative care contact was primarily on the assessment and therapeutic advice in symptom management (70%), assistance in transfer to SPC services (65%), communication and support for families (55%), and decision-making (30%).

### Family Questionnaire

Seventeen out of 20 (85%) family members completed the questionnaire; 59% (*n* = 10) were female, and 41% (*n* = 7) male. Their mean age was 59 years (42–72 years); 47% (*n* = 8) were daughters, 24% (*n* = 4) sons, 18% (*n* = 3) siblings, and 12% (*n* = 2) had other relationships to the patients; 36% (*n* = 5) had been involved as family members of a stroke patient before.

**Figure 1:**
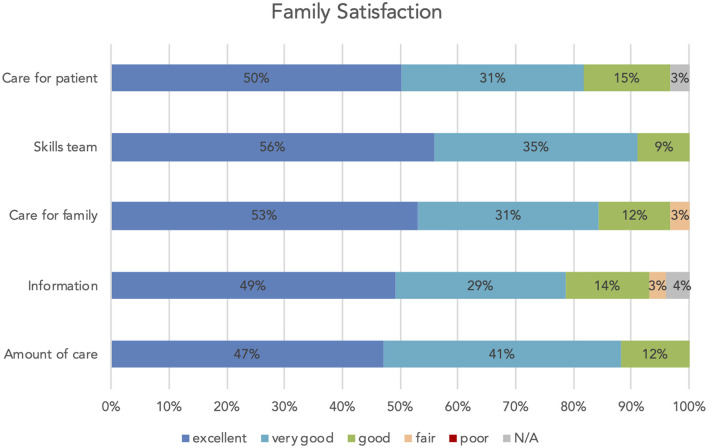
Family satisfaction: care for the patient (concern and caring, management of pain, breathlessness and agitation), skills team (physicians and nurses), care for family (consideration of needs, emotional support, coordination of care, concern and caring), information (frequency, ease of getting, understanding, honesty, completeness and consistency), amount/level of care patient received.

Care for the patient (concern and caring, pain, breathlessness and agitation management); skills and competencies of the stroke unit team (physicians and nurses); care for family members themselves (consideration of needs, emotional support, coordination of care, concern, and caring); and information (frequency, ease of getting, understanding, honesty, completeness, and consistency) were rated excellent, very good, or good by most participants. Most of them were also satisfied with the amount of care the patient received (see Figure 1).

**Figure 2:**
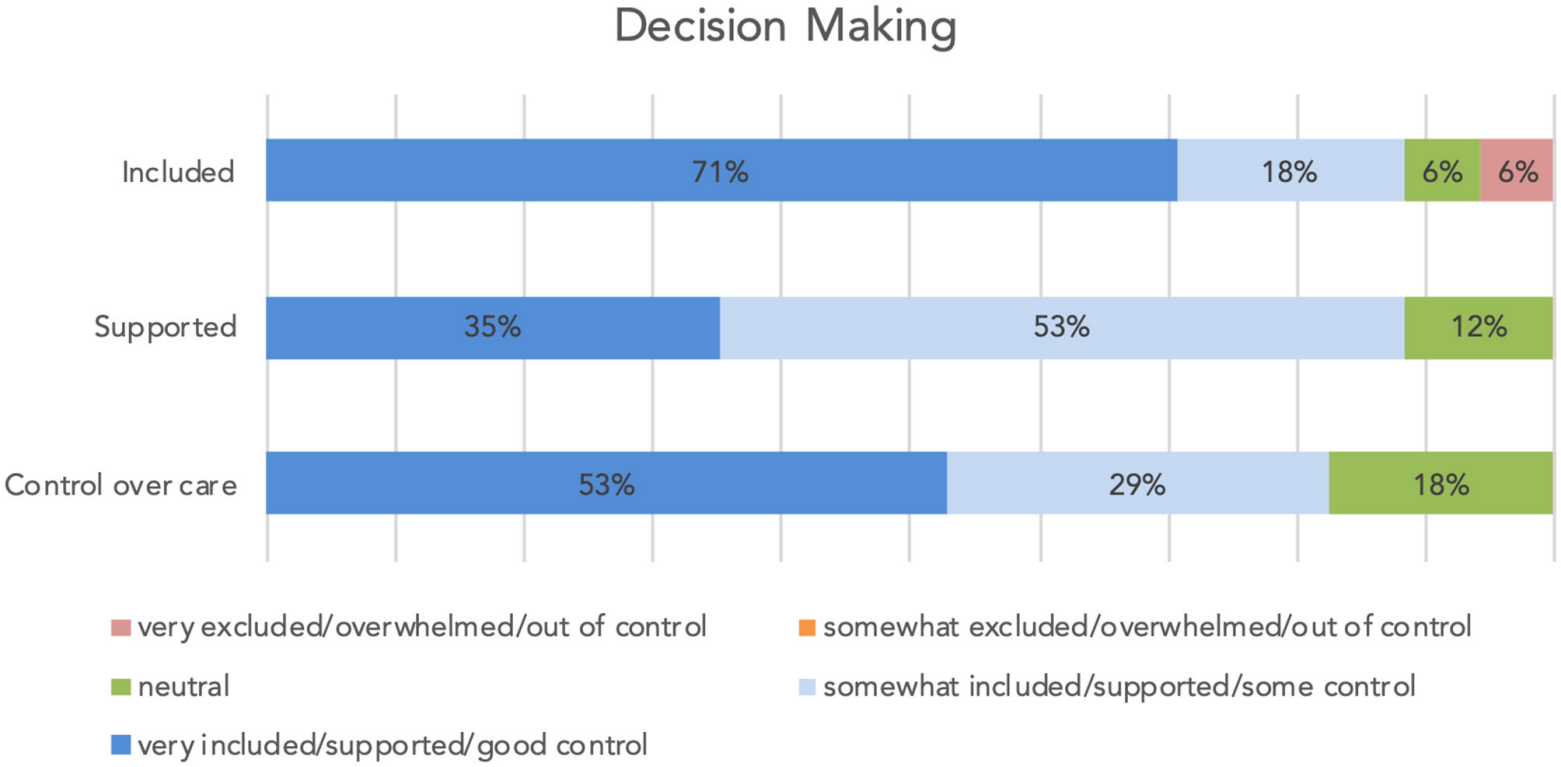
Decision making: included in the decision process, supported during the decision process, control over the care the patient received.

Most participants felt included and supported in decision-making and had the feeling of control over the care their family member received (see Figure 2). The vast majority of the relatives (14 out of 16 participants) felt that the time for addressing concerns and questions during decision-making was adequate; two participants (13%) would have needed more time.

### Team Questionnaire

The stroke team was questioned about their approach to limiting LST; 18 out of 32 team members (56%) completed the survey; 59% (*n* = 10) were nurses and 41% (*n* = 7) were physicians.

57% of the physicians reported needing to deal with the topic of limiting LST at least once a week, and 43% 1–2 times per month.

**Figure 3:**
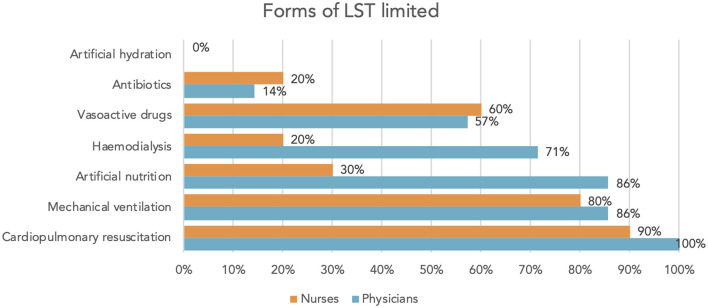
“What were the most common LST withheld from your patients and thus no longer stopped the patient's death?” answers of physicians/nurses.

When asked “*What were the most common LST withheld from your patients and thus no longer stopped the patient's death?*” physicians and nurses reported that CPR or mechanical ventilation was often withheld. None of the participants reported forgoing artificial hydration. Regarding artificial nutrition, the perceptions of nurses and physicians differed: nurses reported withholding artificial nutrition in 30% and physicians in 86% of cases (Figure 3).

**Figure 4:**
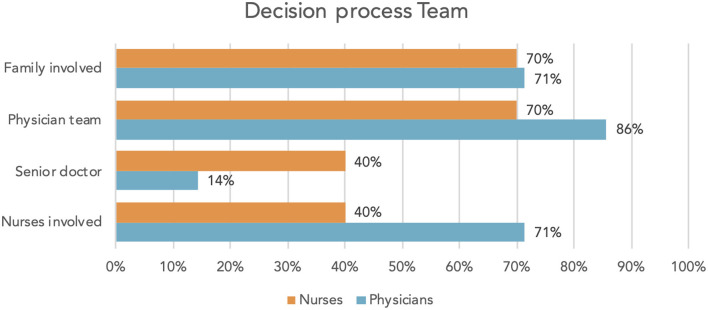
Decision process: “How are decisions about limiting LST made?”.

When questioned about the decision-making process concerning limiting LST, physicians and nurses alike reported the involvement of the patient's family. The decisions were made cooperatively by the physician's team rather than by senior doctors individually. Physicians reported that nurses were involved in 71% of cases, whereas nurses themselves felt involved only in 40% (Figure 4). Satisfaction with decisions (physicians 100%, nurses 90%) and communication (physicians 100%, nurses 80%) was high in both professional groups.

**Figure 5:**
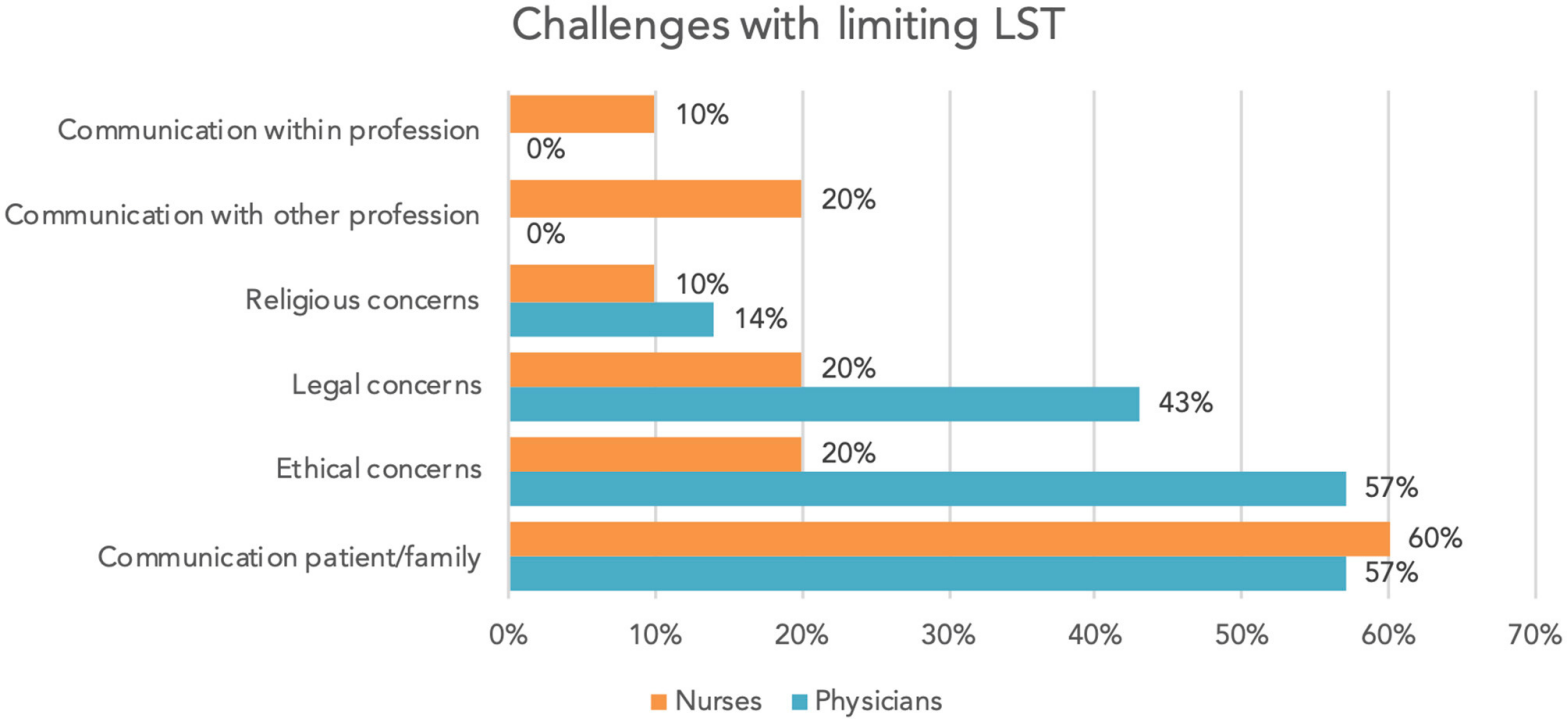
Challenges for team with limiting LST.

Of physicians, 14% felt insufficiently trained and insecure in situations dealing with limiting LST, whereas of nurses, 30% felt insufficiently trained and 10%, insecure. Both groups reported communication with the patient and/or the family challenging. In addition, physicians reported ethical and legal concerns (Figure 5).

Whereas, all involved physicians reported raising the issue of advance directives with patients or relatives, the rate stood only at 50% among the nurses questioned.

Overall, the topic of limiting LST was considered important by both physicians and nurses (100% each) and their need for information proved high (physicians 100%, nurses 90%).

## Discussion

Current recommendations for stroke management in German-speaking countries (Austria, Switzerland, Germany) do not cover palliative care. Our small study is the first evaluation of SPC in stroke patients in Austria. Albeit having been a monocentric and small trial (stroke unit of Barmherzige Schwestern in Ried), we were able to gain some insights into palliative care service for stroke patients. We identified that out of all stroke patients, 4.6% received SPC. This number is consistent with data from the USA (13), but considerably lower than in Australia where 11.4% of all stroke patients received SPC (14).

### Patient Characteristics and Treatment

The patient's average age was 83 (±9 years; mean ± SD) and 65% of them needed preliminarily care. They suffered from severe stroke (mean modified Rankin Scale 5); 80% had cardiac comorbidities and in 45% dementia had been diagnosed before admission for acute stroke. In Canada and the United States, palliative approach is especially recommended for these patients, who have been hit by a severe stroke affecting daily functioning, life quality, and life expectancy as well as having existing significant comorbidities (2, 3).

Acute stroke care is a highly standardized procedure. Data from the USA show that even for patients who were transferred to hospice or died, initial therapy was applied in a timely manner and with high adherence to stroke process measures (15). This is again comparable with our data. Although our study population would have profited less from intravenous thrombolysis due to their age, existing comorbidities and preliminary need for care, intravenous thrombolysis was administered in 60% of cases.

The death of 12 patients in our study occurred on average 11 (±11) days after admission and 6 days after setting the therapy goal to comfort terminal care. The mean PREMISE score predicting mortality within the first week after admission to the stroke unit (12) was 8 (±2), which would imply a mortality of 19% within the first week. Since 25% (*n* = 5) of our patients died within this period, the usefulness of this score can be seen, even in this small sample. The prognostic accuracy of the PREMISE score in patients with acute ischemic stroke has been also demonstrated in a larger cohort in Greece (16). Therefore, its use might prove a valuable indicator for the need of palliative care support. However, the value of prognostic models has not been established for post-stroke end-of-life treatment decisions (3).

### Decision-Making

In severe stroke, prognosis is frequently unclear, as patients suffer acute neurological deterioration and are often unable to communicate and make decisions for themselves. In this setting, clinicians and families frequently need to make treatment decisions. Shared decision-making is an approach where patients, families, and clinicians consider patient's values and preferences alongside the best medical evidence and cooperate to make the best decision for a given patient in a specific scenario. This approach can be applied to every decision within stroke care (17).

In our sample, only three patients were capable of making decisions for themselves and all of them refused intensive care measures and CPR.

Two patients (10%) had preexisting advance directives on admission, which is average for Austria where about 8% of over 70-year-olds have an advance directive (18). Although the relevant law was established in Austria already in 2006, some medical professionals are still insecure about the completion and application of advance directives (18) and avoid the topic. Enhanced knowledge (19) and the role of decision-maker could be the reasons why advance directives were addressed by all of physicians but only by 50% of the nurses who participated in our research.

Due to the severity of stroke or preexisting comorbidities, for example, dementia, most decisions in our research were surrogate decisions. Decisions about limiting LST were reported to having been made in cooperation between the physician's team and the patient's family. Families were satisfied with the information provided and felt included and supported in the decision-making process.

In this research, physicians stated that in 71% of cases, nurses were involved in the decision-making process. Nurses themselves, however, felt being involved in only 40% of cases. Our data did not reveal the reasons for this disparity. Although the involvement of nurses in end-of-life decision-making for patients with acute stroke influences neurologist's intensivist practice and behavior and may help them (20), nurses are rarely involved because of the lack of awareness, knowledge, and time as well as hierarchical reasons (11, 21). On the other hand, nurses are highly involved in executing these decisions. This might be identified as a relevant risk factor for burnout in ICU personnel (22). At the same time, overall satisfaction with decisions and communication concerning limitation of LST was high in both professions in this team.

### Limiting Life-Sustaining Therapies

As treatment restrictions are independently associated with mortality (23), decisions on withholding or withdrawal of life-sustaining treatments should be taken with great caution (6). Yet, this highly demanding procedure is common in intensive care units. Physicians feel confronted with the topic more frequently than nurses.

All of our patients had individual therapeutic goals with directives for gradual forgoing of treatment completed at an early stage. Despite these time pressures on decisions concerning therapeutic goals, 88% of the questioned family members stated that they had adequate time to have their concerns addressed and questions answered during the decision-making process; 88% of the questioned family members were completely satisfied or very satisfied with the level or amount of health care provided to their family member.

In our research, CPR proved the most limited form of LST mentioned by nurses and physicians. All of our analyzed patients had a Do Not Resuscitate order. In general, 18% of in-hospital cardiac arrest patients survive to discharge and age over 70: altered mental status, need for assistance in every day activities, and admission for medical non-cardiac diagnosis are considered to be in strong correlation with the failure to survive to discharge (24). Evidence for the outcome of CPR in stroke patients is lacking, yet forgoing CPR in stroke is common (25). 35% of the sample received antibiotic therapy and thus it would seem that the restriction of CPR was considered differently to other LST (3).

In the researched setting that does not offer mechanical ventilation, forgoing ventilation was reported more often than in ICUs (11). In contrast, withdrawing mechanical ventilation is regarded more difficult since it is associated with legal and ethical concerns (26) and needs highly specialized palliative care for sufficient symptom control.

Stroke patients are prone to malnutrition, dehydration, and aspiration pneumonia due to dysphagia, impaired consciousness, perception deficits, and cognitive dysfunction. When dysphagia is considered prolonged, percutaneous endoscopic gastrostomy (PEG) is recommended (27) but it is also associated with persisting impairment of swallowing and mobility and a mortality of 66% after 2 years (28). Decisions about nutrition can be highly emotional for families and can result in conflicts with treatment teams (29). Forgoing PEG placement and artificial nutrition is more frequent in stroke patients admitted to palliative care (13, 30) and was reported by 86% of the physicians questioned in our research. Nurses reported the limitation of artificial nutrition less frequently than doctors (30 vs. 86%), as shown in previous research (31).

Although forgoing artificial hydration in dying patients is recommended when no benefits are expected (32), all team members stated that hydration is never withheld. This has been also documented in previous reports (30). Some countries or cultures consider hydration as a basic measure that cannot be withheld (32). As well as personal beliefs, religious and cultural considerations have an important role in this decision.

Our data show that the topic of limiting LST is important for nurses as well as physicians and the need for information is high. Compared to data from German ICUs (11), our staff felt less insecure about applying LST, felt better trained, and had less fears for legal consequences. Ethical policies and consultations implemented in our hospital appear to facilitate decision making (11).

To structure, de-emotionalize, and make decisions on limiting LST reproducible for others, standardized documentation is recommended (11, 33). Whereas, German ICU personnel stated that 32% of cases would have no written documentation about Do Not Resuscitate orders (11), the implemented form for documentation of resuscitation status was used in every patient analyzed. For further steps of forgoing therapy (Do Not Escalate DNE, Comfort Terminal Care CTC), our hospital has no standardized documentation protocols, leading to reduced documentation on these.

### Palliative Care in the Stroke Unit

Palliative care needs of patients with severe stroke and their families are high requiring complex decision-making, aligning treatment with goals and symptom control (3). Uncertain prognosis, communication, and quality of life are specific issues for palliative care in the Neuro-ICU (34). Frequently, family members are the main recipients of SPC (35).

In the researched setting, the focus of SPC consultations was mainly on the assessment and therapeutic advice in symptom management, followed by assistance in transfer to SPC services.

Assistance in discussing and clarifying care goals is a common indication for palliative care consultations (34, 35). Indeed, in palliative care consultations a lot of time is spent on discussing prognosis, family's understanding of prognosis, and exploring patient's and family's values, whereas neurologists and intensivists are in charge of prognostication (35). Interestingly, in our sample, assistance in discussing and clarifying goals was part of the consultations in only 30% of cases. The limited availability of the SPC team, who are available only during day time working hours, may have influenced the neurologists to develop palliative care skills. Further research is needed to look at this area.

SPC services are often used when the care team believes that issues regarding the withdrawal of LST are the focus of a patient's management (34). Our data shows that in the context of limiting LST, communication with patients and/or their families was the biggest challenge for stroke unit professionals. As shown before (35), communication and support for families were a frequent part of SPC consultations in the researched setting.

### Limitations

Our study has several limitations, above all, the single-center trial and, thus, the small number of patients as well as team members. Expanding it to other Austria stroke units would give a better overview about clinical practice and the level of integration of palliative care in the country.

Creutzfeldt et al. demonstrated that a brief palliative care needs screening tool had the potential to improve care for patients and their families (36, 37). In our research, the involvement of SPC was based on individual decisions, which, according to a recent survey (34) was the preferred way of access also among US neurointensivists. With the chosen approach, some patients and families with palliative needs may not have been identified.

Our sample included only Caucasian people, which reflects the ethnic structure in our region.

## Conclusion

Although palliative care is a recognized part of stroke care (38) this is the first study in Austria to examine an approach with early and close cooperation with SPC services, resulting in setting patient-centered therapeutic goals early in the acute phase of severe stroke. While acknowledging the small sample size, families' satisfaction with the care delivered to the patients, including the level or amount of health care, was high. Similarly, families were highly satisfied with decision-making processes as well as with information and support received. Furthermore, we could gain information about team decision-making process, especially concerning limiting LST, in the context of acute stroke care.

## Data Availability Statement

The raw data supporting the conclusions of this article will be made available by the authors, without undue reservation.

## Ethics Statement

The studies involving human participants were reviewed and approved by Ethics committee of Krankenhaus Barmherzige Schwestern Ried im Innkreis. Written informed consent for participation was not required for this study in accordance with the national legislation and the institutional requirements.

## Author Contributions

RR and SL contributed to conception and design of the study based on former research by KA. RR organized the database, performed the statistical analysis, and wrote the first draft of the manuscript. All authors contributed to manuscript revision, read, and approved the submitted version.

## Conflict of Interest

The authors declare that the research was conducted in the absence of any commercial or financial relationships that could be construed as a potential conflict of interest. The Handling Editor declared a past co-authorship with one of the authors, SL.

## Publisher's Note

All claims expressed in this article are solely those of the authors and do not necessarily represent those of their affiliated organizations, or those of the publisher, the editors and the reviewers. Any product that may be evaluated in this article, or claim that may be made by its manufacturer, is not guaranteed or endorsed by the publisher.

## Abbreviations

CPR: cardiopulmonary resuscitation
DNR: Do Not Resuscitate
ICU: intensive care unit
LST: life-sustaining therapy/therapies
SPC: specialist palliative care.
